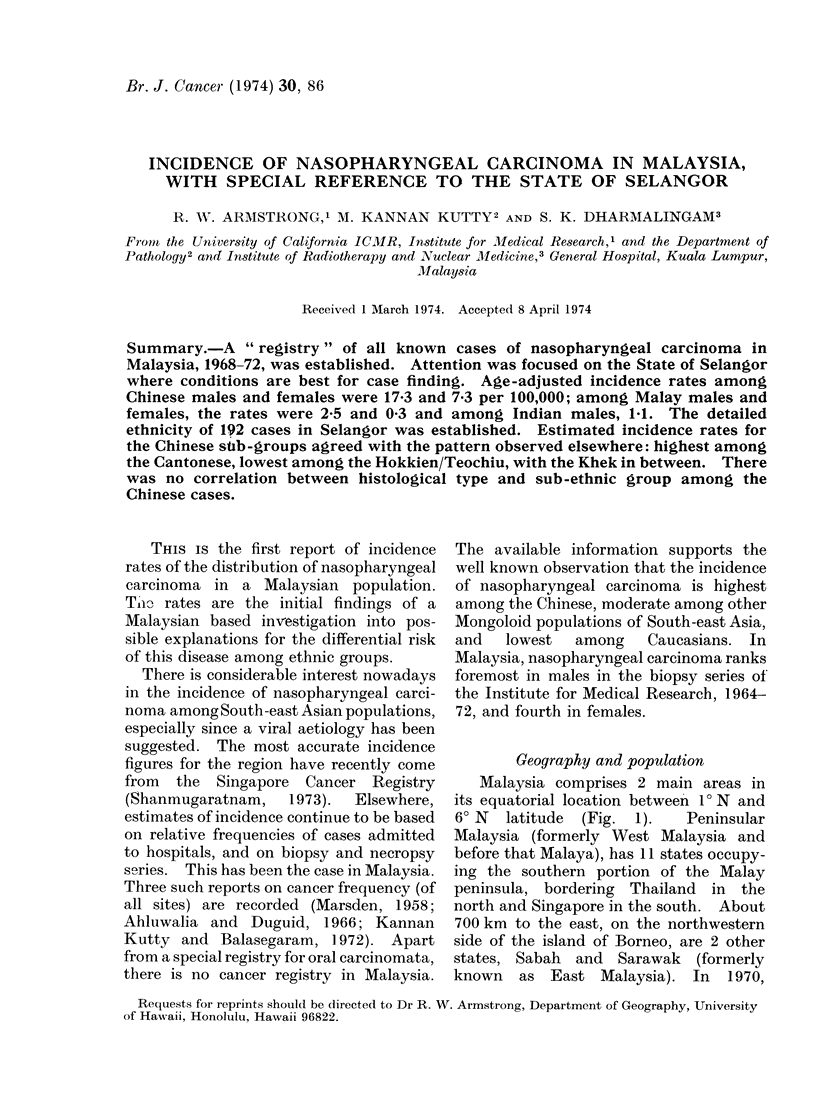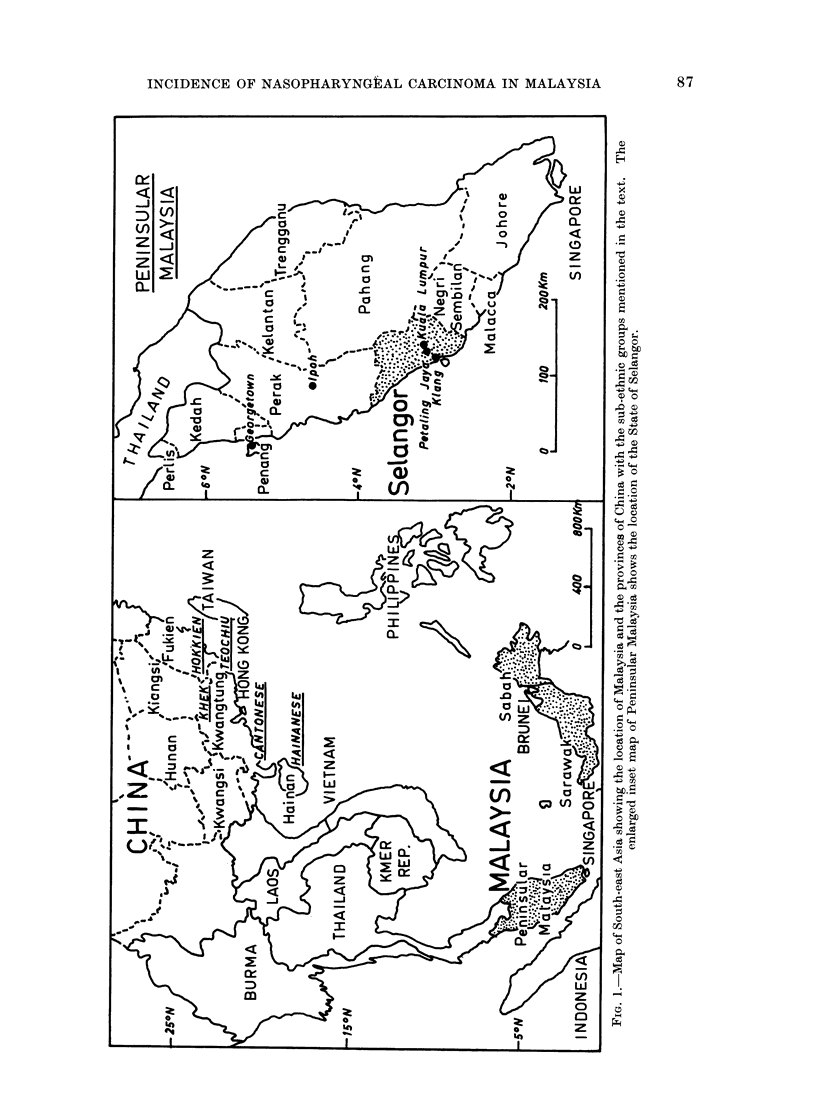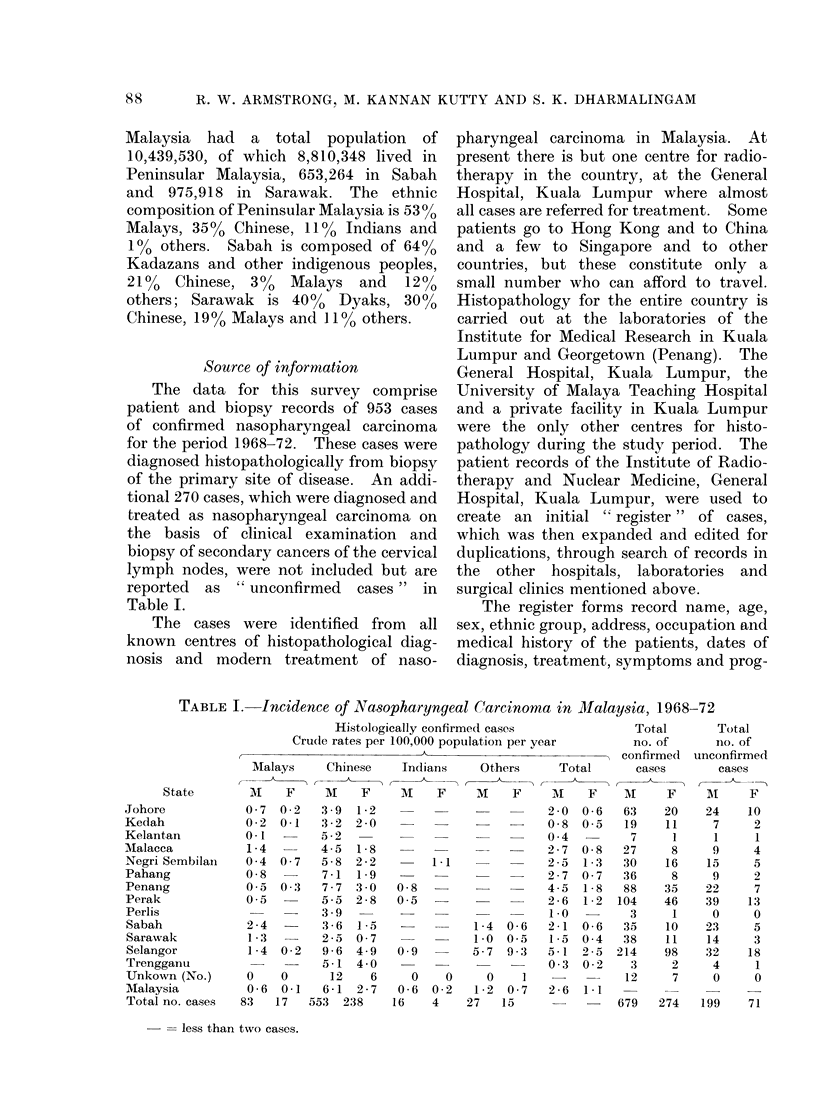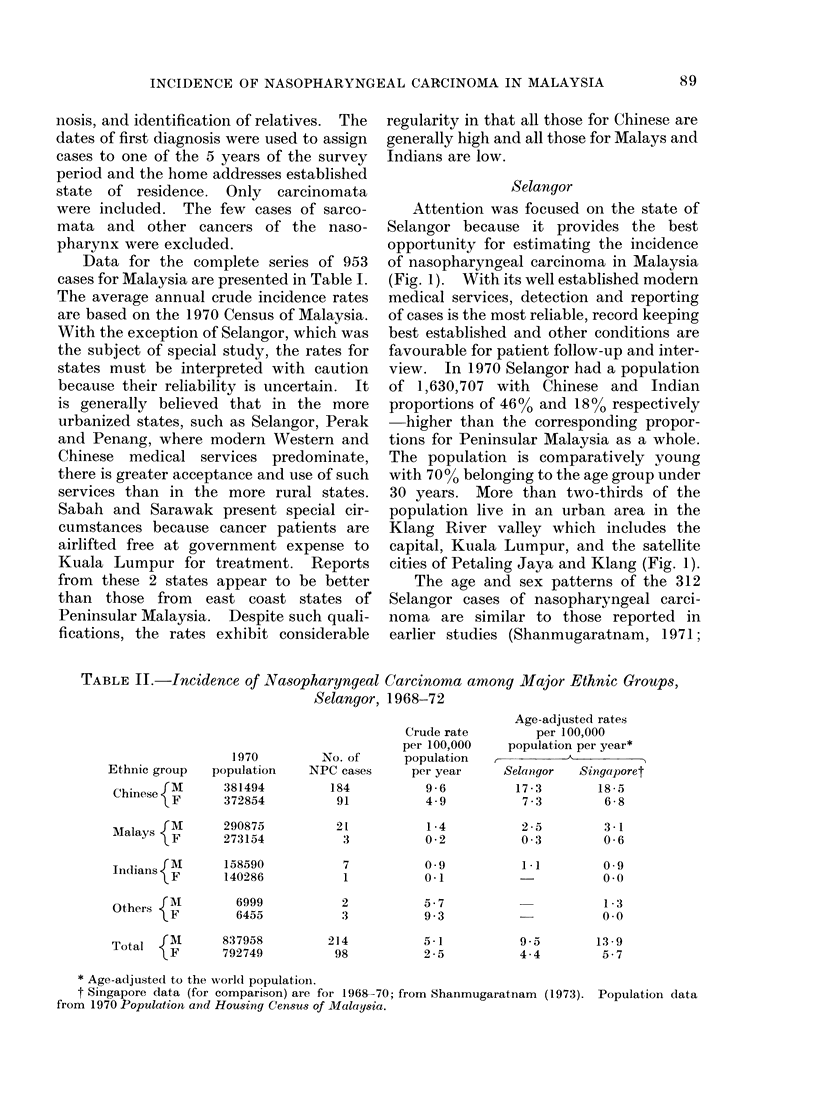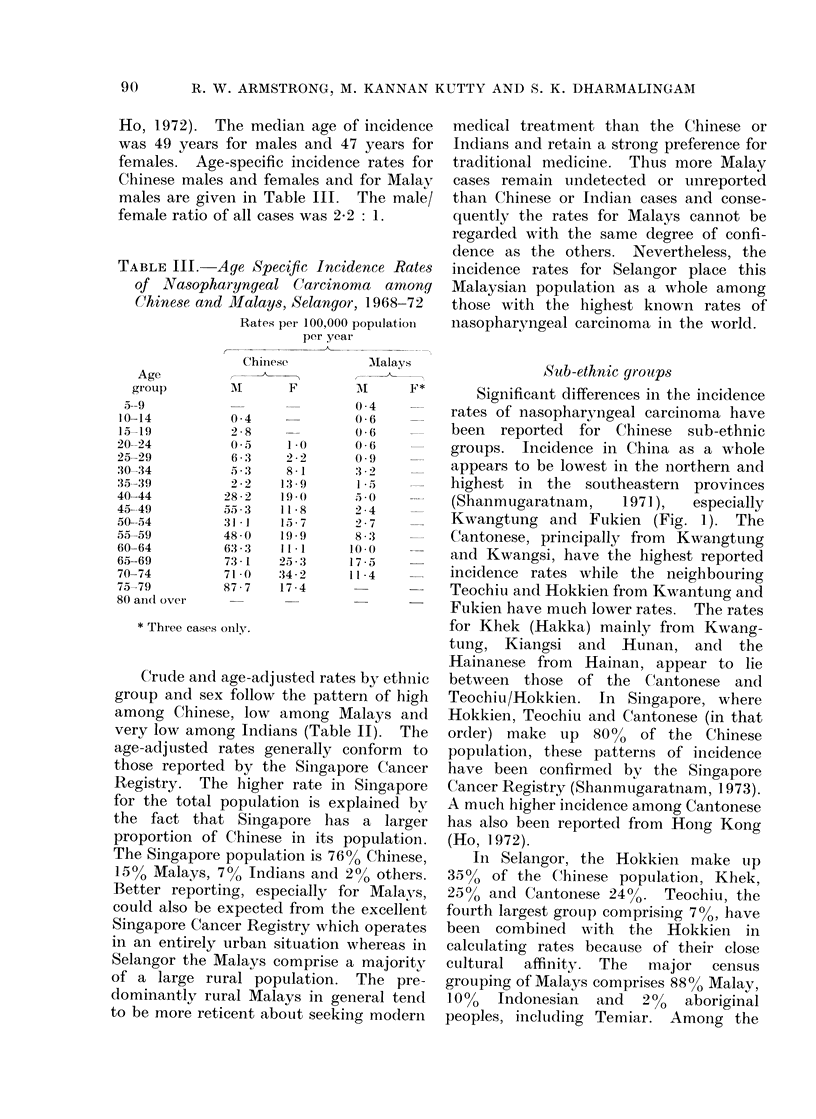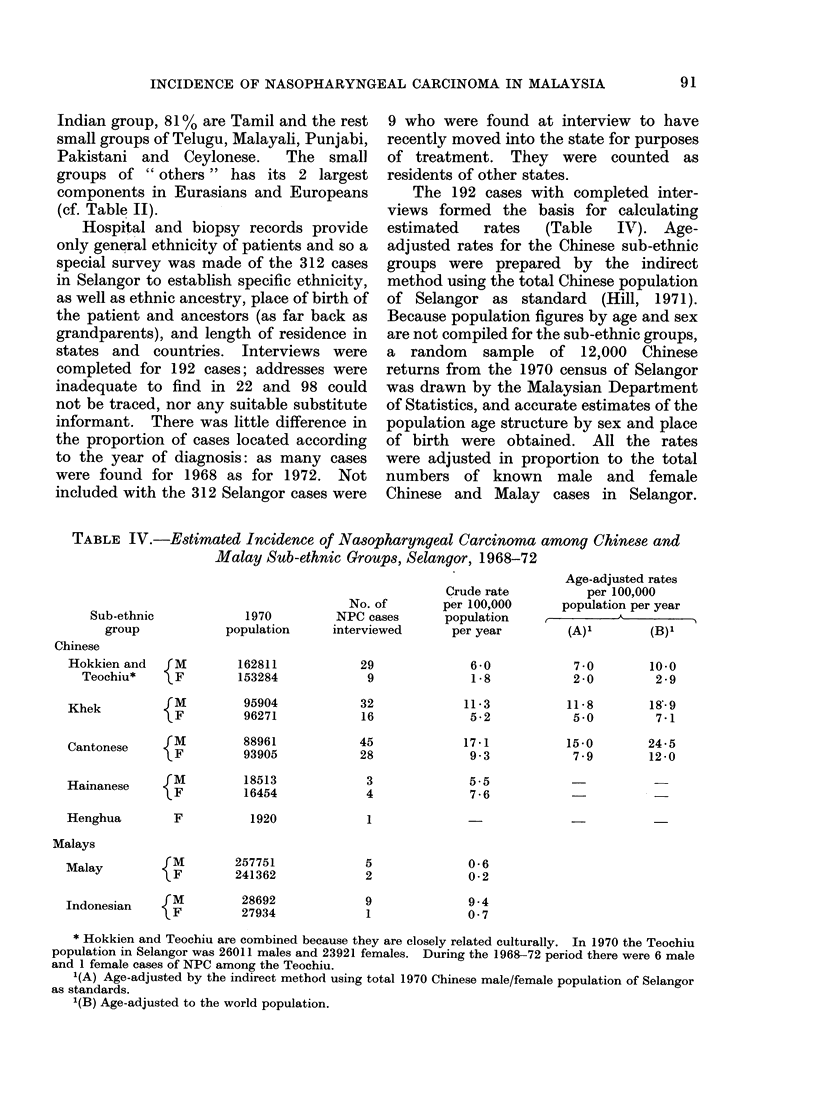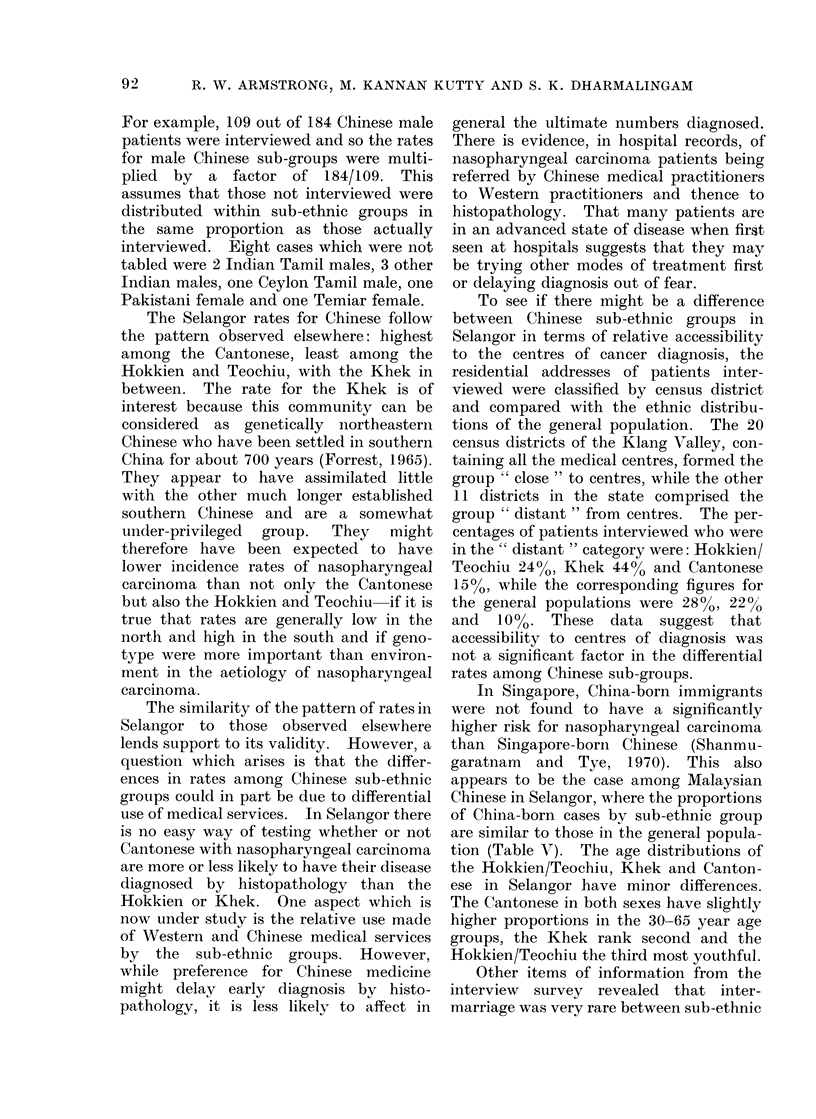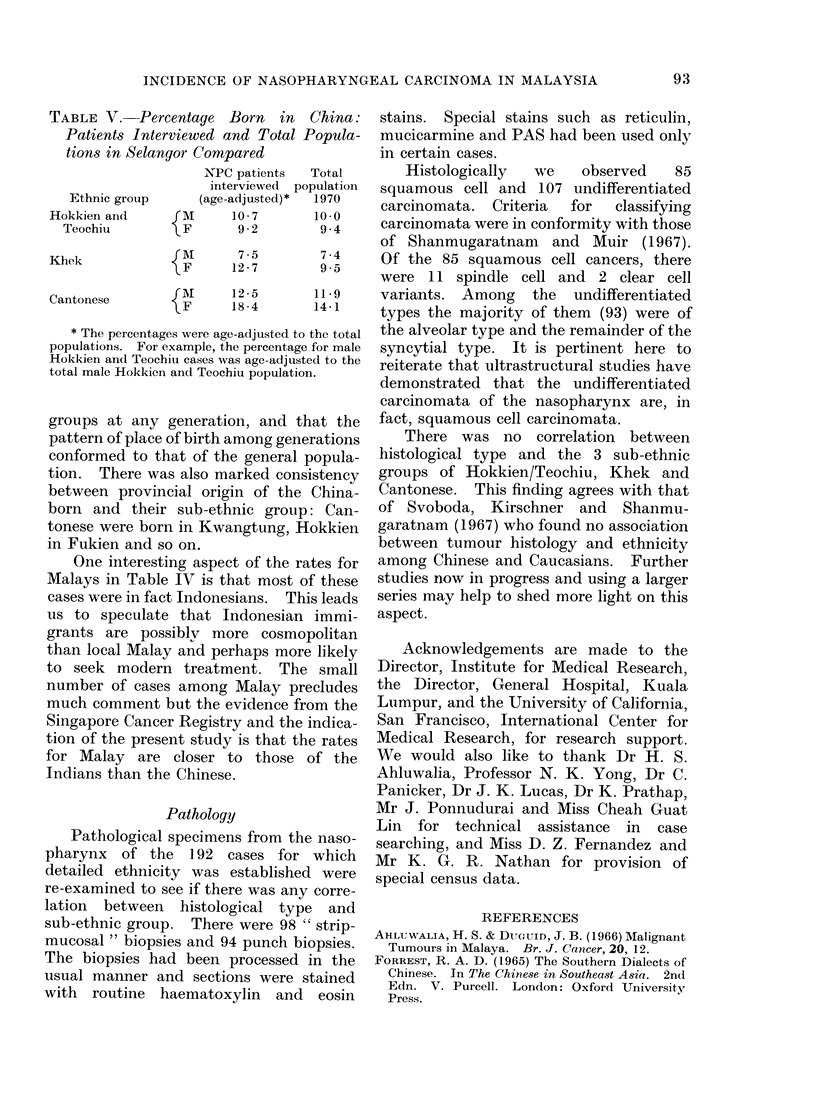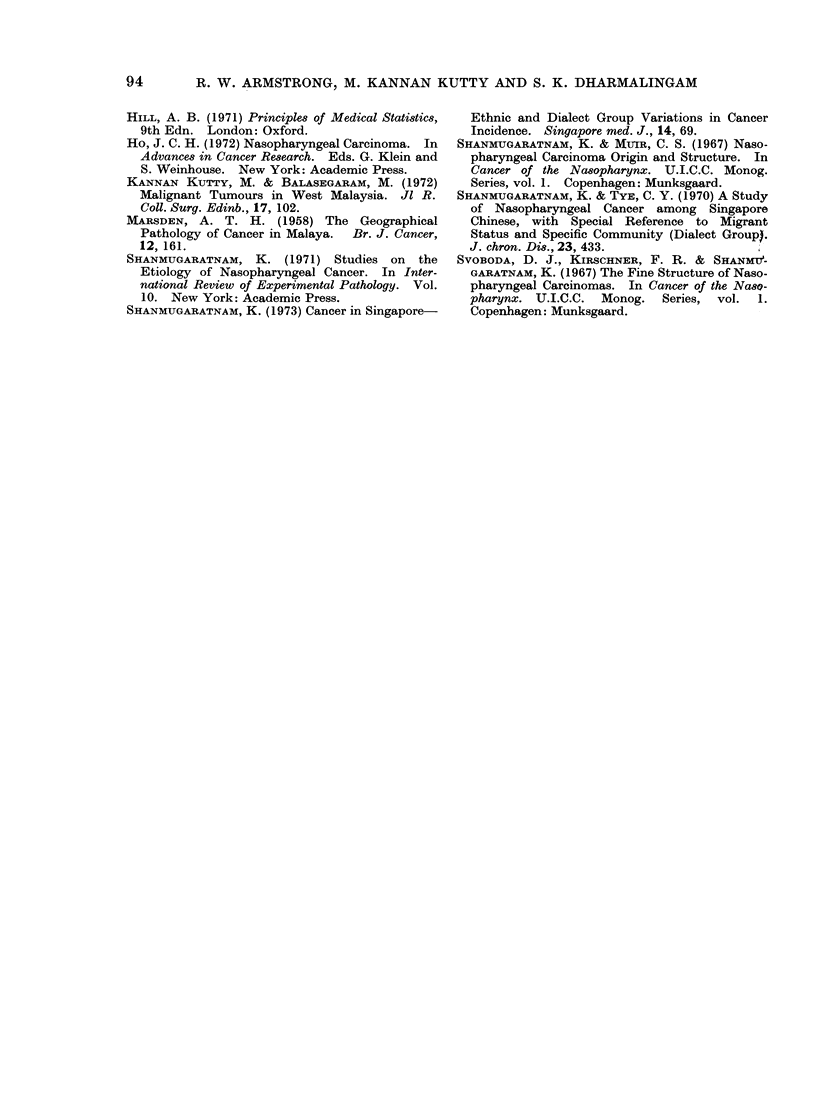# Incidence of Nasopharyngeal Carcinoma in Malaysia, with Special Reference to the State of Selangor

**DOI:** 10.1038/bjc.1974.116

**Published:** 1974-07

**Authors:** R. W. Armstrong, M. Kutty, S. K. Dharmalingam

## Abstract

A “registry” of all known cases of nasopharyngeal carcinoma in Malaysia, 1968-72, was established. Attention was focused on the State of Selangor where conditions are best for case finding. Age-adjusted incidence rates among Chinese males and females were 17·3 and 7·3 per 100,000; among Malay males and females, the rates were 2·5 and 0·3 and among Indian males, 1·1. The detailed ethnicity of 192 cases in Selangor was established. Estimated incidence rates for the Chinese sub-groups agreed with the pattern observed elsewhere: highest among the Cantonese, lowest among the Hokkien/Teochiu, with the Khek in between. There was no correlation between histological type and sub-ethnic group among the Chinese cases.


Br. J. Cancer (1974) 30, 86

### INCIDENCE OF NASOPHARYNGEAL CARCINOMA IN MALAYSIA, WITH SPECIAL REFERENCE TO THE STATE OF SELANGOR

R. W. ARMSTRONG,[1] M. KANNAN KUTTY[2] AND S. K. DHARMALINGAM[3]

*From the University of California ICMR, Institute for Medical Research,[1] and the Department of Pathology[2] and Institute of Radiotherapy and Nuclear Medicine,[3] General Hospital, Kuala Lumpur, Malaysia*

Received 1 March 1974. Accepted 8 April 1974

**Summary.—A "registry" of all known cases of nasopharyngeal carcinoma in Malaysia, 1968–72, was established. Attention was focused on the State of Selangor where conditions are best for case finding. Age-adjusted incidence rates among Chinese males and females were 17·3 and 7·3 per 100,000; among Malay males and females, the rates were 2·5 and 0·3 and among Indian males, 1·1. The detailed ethnicity of 192 cases in Selangor was established. Estimated incidence rates for the Chinese sub-groups agreed with the pattern observed elsewhere: highest among the Cantonese, lowest among the Hokkien/Teochiu, with the Khek in between. There was no correlation between histological type and sub-ethnic group among the Chinese cases.**

THIS IS the first report of incidence rates of the distribution of nasopharyngeal carcinoma in a Malaysian population. The rates are the initial findings of a Malaysian based investigation into possible explanations for the differential risk of this disease among ethnic groups.

There is considerable interest nowadays in the incidence of nasopharyngeal carcinoma among South-east Asian populations, especially since a viral aetiology has been suggested. The most accurate incidence figures for the region have recently come from the Singapore Cancer Registry (Shanmugaratnam, 1973). Elsewhere, estimates of incidence continue to be based on relative frequencies of cases admitted to hospitals, and on biopsy and necropsy series. This has been the case in Malaysia. Three such reports on cancer frequency (of all sites) are recorded (Marsden, 1958; Ahluwalia and Duguid, 1966; Kannan Kutty and Balasegaram, 1972). Apart from a special registry for oral carcinomata, there is no cancer registry in Malaysia. The available information supports the well known observation that the incidence of nasopharyngeal carcinoma is highest among the Chinese, moderate among other Mongoloid populations of South-east Asia, and lowest among Caucasians. In Malaysia, nasopharyngeal carcinoma ranks foremost in males in the biopsy series of the Institute for Medical Research, 1964–72, and fourth in females.

### *Geography and population*

Malaysia comprises 2 main areas in its equatorial location between 1° N and 6° N latitude (Fig. 1). Peninsular Malaysia (formerly West Malaysia and before that Malaya), has 11 states occupying the southern portion of the Malay peninsula, bordering Thailand in the north and Singapore in the south. About 700 km to the east, on the northwestern side of the island of Borneo, are 2 other states, Sabah and Sarawak (formerly known as East Malaysia). In 1970,

Requests for reprints should be directed to Dr R. W. Armstrong, Department of Geography, University of Hawaii, Honolulu, Hawaii 96822.

FIG. 1.—Map of South-east Asia showing the location of Malaysia and the provinces of China with the sub-ethnic groups mentioned in the text. The enlarged inset map of Peninsular Malaysia shows the location of the State of Selangor.

Malaysia had a total population of 10,439,530, of which 8,810,348 lived in Peninsular Malaysia, 653,264 in Sabah and 975,918 in Sarawak. The ethnic composition of Peninsular Malaysia is 53% Malays, 35% Chinese, 11% Indians and 1% others. Sabah is composed of 64% Kadazans and other indigenous peoples, 21% Chinese, 3% Malays and 12% others; Sarawak is 40% Dyaks, 30% Chinese, 19% Malays and 11% others.

### *Source of information*

The data for this survey comprise patient and biopsy records of 953 cases of confirmed nasopharyngeal carcinoma for the period 1968–72. These cases were diagnosed histopathologically from biopsy of the primary site of disease. An additional 270 cases, which were diagnosed and treated as nasopharyngeal carcinoma on the basis of clinical examination and biopsy of secondary cancers of the cervical lymph nodes, were not included but are reported as " unconfirmed cases " in Table I.

The cases were identified from all known centres of histopathological diagnosis and modern treatment of nasopharyngeal carcinoma in Malaysia. At present there is but one centre for radiotherapy in the country, at the General Hospital, Kuala Lumpur where almost all cases are referred for treatment. Some patients go to Hong Kong and to China and a few to Singapore and to other countries, but these constitute only a small number who can afford to travel. Histopathology for the entire country is carried out at the laboratories of the Institute for Medical Research in Kuala Lumpur and Georgetown (Penang). The General Hospital, Kuala Lumpur, the University of Malaya Teaching Hospital and a private facility in Kuala Lumpur were the only other centres for histopathology during the study period. The patient records of the Institute of Radiotherapy and Nuclear Medicine, General Hospital, Kuala Lumpur, were used to create an initial " register " of cases, which was then expanded and edited for duplications, through search of records in the other hospitals, laboratories and surgical clinics mentioned above.

The register forms record name, age, sex, ethnic group, address, occupation and medical history of the patients, dates of diagnosis, treatment, symptoms and prog-

TABLE I.—*Incidence of Nasopharyngeal Carcinoma in Malaysia*, 1968–72

| | Histologically confirmed cases Crude rates per 100,000 population per year | | | | | | | | | | Total no. of confirmed cases | | Total no. of unconfirmed cases | |
|---|---|---|---|---|---|---|---|---|---|---|---|---|---|---|
| | Malays | | Chinese | | Indians | | Others | | Total | | | | | |
| State | M | F | M | F | M | F | M | F | M | F | M | F | M | F |
| Johore | 0·7 | 0·2 | 3·9 | 1·2 | — | — | — | — | 2·0 | 0·6 | 63 | 20 | 24 | 10 |
| Kedah | 0·2 | 0·1 | 3·2 | 2·0 | — | — | — | — | 0·8 | 0·5 | 19 | 11 | 7 | 2 |
| Kelantan | 0·1 | — | 5·2 | — | — | — | — | — | 0·4 | — | 7 | 1 | 1 | 1 |
| Malacca | 1·4 | — | 4·5 | 1·8 | — | — | — | — | 2·7 | 0·8 | 27 | 8 | 9 | 4 |
| Negri Sembilan | 0·4 | 0·7 | 5·8 | 2·2 | — | 1·1 | — | — | 2·5 | 1·3 | 30 | 16 | 15 | 5 |
| Pahang | 0·8 | — | 7·1 | 1·9 | — | — | — | — | 2·7 | 0·7 | 36 | 8 | 9 | 2 |
| Penang | 0·5 | 0·3 | 7·7 | 3·0 | 0·8 | — | — | — | 4·5 | 1·8 | 88 | 35 | 22 | 7 |
| Perak | 0·5 | — | 5·5 | 2·8 | 0·5 | — | — | — | 2·6 | 1·2 | 104 | 46 | 39 | 13 |
| Perlis | — | — | 3·9 | — | — | — | — | — | 1·0 | — | 3 | 1 | 0 | 0 |
| Sabah | 2·4 | — | 3·6 | 1·5 | — | — | 1·4 | 0·6 | 2·1 | 0·6 | 35 | 10 | 23 | 5 |
| Sarawak | 1·3 | — | 2·5 | 0·7 | — | — | 1·0 | 0·5 | 1·5 | 0·4 | 38 | 11 | 14 | 3 |
| Selangor | 1·4 | 0·2 | 9·6 | 4·9 | 0·9 | — | 5·7 | 9·3 | 5·1 | 2·5 | 214 | 98 | 32 | 18 |
| Trengganu | — | — | 5·1 | 4·0 | — | — | — | — | 0·3 | 0·2 | 3 | 2 | 4 | 1 |
| Unkown (No.) | 0 | 0 | 12 | 6 | 0 | 0 | 0 | 1 | — | — | 12 | 7 | 0 | 0 |
| Malaysia | 0·6 | 0·1 | 6·1 | 2·7 | 0·6 | 0·2 | 1·2 | 0·7 | 2·6 | 1·1 | — | — | — | — |
| Total no. cases | 83 | 17 | 553 | 238 | 16 | 4 | 27 | 15 | — | — | 679 | 274 | 199 | 71 |

— = less than two cases.

nosis, and identification of relatives. The dates of first diagnosis were used to assign cases to one of the 5 years of the survey period and the home addresses established state of residence. Only carcinomata were included. The few cases of sarcomata and other cancers of the nasopharynx were excluded.

Data for the complete series of 953 cases for Malaysia are presented in Table I. The average annual crude incidence rates are based on the 1970 Census of Malaysia. With the exception of Selangor, which was the subject of special study, the rates for states must be interpreted with caution because their reliability is uncertain. It is generally believed that in the more urbanized states, such as Selangor, Perak and Penang, where modern Western and Chinese medical services predominate, there is greater acceptance and use of such services than in the more rural states. Sabah and Sarawak present special circumstances because cancer patients are airlifted free at government expense to Kuala Lumpur for treatment. Reports from these 2 states appear to be better than those from east coast states of Peninsular Malaysia. Despite such qualifications, the rates exhibit considerable regularity in that all those for Chinese are generally high and all those for Malays and Indians are low.

*Selangor*

Attention was focused on the state of Selangor because it provides the best opportunity for estimating the incidence of nasopharyngeal carcinoma in Malaysia (Fig. 1). With its well established modern medical services, detection and reporting of cases is the most reliable, record keeping best established and other conditions are favourable for patient follow-up and interview. In 1970 Selangor had a population of 1,630,707 with Chinese and Indian proportions of 46% and 18% respectively —higher than the corresponding proportions for Peninsular Malaysia as a whole. The population is comparatively young with 70% belonging to the age group under 30 years. More than two-thirds of the population live in an urban area in the Klang River valley which includes the capital, Kuala Lumpur, and the satellite cities of Petaling Jaya and Klang (Fig. 1).

The age and sex patterns of the 312 Selangor cases of nasopharyngeal carcinoma are similar to those reported in earlier studies (Shanmugaratnam, 1971;

TABLE II.—*Incidence of Nasopharyngeal Carcinoma among Major Ethnic Groups, Selangor*, 1968–72

| Ethnic group | | 1970 population | No. of NPC cases | Crude rate per 100,000 population per year | Age-adjusted rates per 100,000 population per year* | |
|---|---|---|---|---|---|---|
| | | | | | *Selangor* | *Singapore*† |
| Chinese | M | 381494 | 184 | 9·6 | 17·3 | 18·5 |
| | F | 372854 | 91 | 4·9 | 7·3 | 6·8 |
| Malays | M | 290875 | 21 | 1·4 | 2·5 | 3·1 |
| | F | 273154 | 3 | 0·2 | 0·3 | 0·6 |
| Indians | M | 158590 | 7 | 0·9 | 1·1 | 0·9 |
| | F | 140286 | 1 | 0·1 | — | 0·0 |
| Others | M | 6999 | 2 | 5·7 | — | 1·3 |
| | F | 6455 | 3 | 9·3 | — | 0·0 |
| Total | M | 837958 | 214 | 5·1 | 9·5 | 13·9 |
| | F | 792749 | 98 | 2·5 | 4·4 | 5·7 |

* Age-adjusted to the world population.

† Singapore data (for comparison) are for 1968–70; from Shanmugaratnam (1973). Population data from 1970 *Population and Housing Census of Malaysia.*

Ho, 1972). The median age of incidence was 49 years for males and 47 years for females. Age-specific incidence rates for Chinese males and females and for Malay males are given in Table III. The male/female ratio of all cases was 2·2 : 1.

TABLE III.—*Age Specific Incidence Rates of Nasopharyngeal Carcinoma among Chinese and Malays, Selangor,* 1968–72

| Age group | Chinese M | Chinese F | Malays M | Malays F* |
|---|---|---|---|---|
| | Rates per 100,000 population per year | | | |
| 5–9 | — | — | 0·4 | — |
| 10–14 | 0·4 | — | 0·6 | — |
| 15–19 | 2·8 | — | 0·6 | — |
| 20–24 | 0·5 | 1·0 | 0·6 | — |
| 25–29 | 6·3 | 2·2 | 0·9 | — |
| 30–34 | 5·3 | 8·1 | 3·2 | — |
| 35–39 | 2·2 | 13·9 | 1·5 | — |
| 40–44 | 28·2 | 19·0 | 5·0 | — |
| 45–49 | 55·3 | 11·8 | 2·4 | — |
| 50–54 | 31·1 | 15·7 | 2·7 | — |
| 55–59 | 48·0 | 19·9 | 8·3 | — |
| 60–64 | 63·3 | 11·1 | 10·0 | — |
| 65–69 | 73·1 | 25·3 | 17·5 | — |
| 70–74 | 71·0 | 34·2 | 11·4 | — |
| 75–79 | 87·7 | 17·4 | — | — |
| 80 and over | — | — | — | — |

* Three cases only.

Crude and age-adjusted rates by ethnic group and sex follow the pattern of high among Chinese, low among Malays and very low among Indians (Table II). The age-adjusted rates generally conform to those reported by the Singapore Cancer Registry. The higher rate in Singapore for the total population is explained by the fact that Singapore has a larger proportion of Chinese in its population. The Singapore population is 76% Chinese, 15% Malays, 7% Indians and 2% others. Better reporting, especially for Malays, could also be expected from the excellent Singapore Cancer Registry which operates in an entirely urban situation whereas in Selangor the Malays comprise a majority of a large rural population. The predominantly rural Malays in general tend to be more reticent about seeking modern medical treatment than the Chinese or Indians and retain a strong preference for traditional medicine. Thus more Malay cases remain undetected or unreported than Chinese or Indian cases and consequently the rates for Malays cannot be regarded with the same degree of confidence as the others. Nevertheless, the incidence rates for Selangor place this Malaysian population as a whole among those with the highest known rates of nasopharyngeal carcinoma in the world.

### *Sub-ethnic groups*

Significant differences in the incidence rates of nasopharyngeal carcinoma have been reported for Chinese sub-ethnic groups. Incidence in China as a whole appears to be lowest in the northern and highest in the southeastern provinces (Shanmugaratnam, 1971), especially Kwangtung and Fukien (Fig. 1). The Cantonese, principally from Kwangtung and Kwangsi, have the highest reported incidence rates while the neighbouring Teochiu and Hokkien from Kwantung and Fukien have much lower rates. The rates for Khek (Hakka) mainly from Kwangtung, Kiangsi and Hunan, and the Hainanese from Hainan, appear to lie between those of the Cantonese and Teochiu/Hokkien. In Singapore, where Hokkien, Teochiu and Cantonese (in that order) make up 80% of the Chinese population, these patterns of incidence have been confirmed by the Singapore Cancer Registry (Shanmugaratnam, 1973). A much higher incidence among Cantonese has also been reported from Hong Kong (Ho, 1972).

In Selangor, the Hokkien make up 35% of the Chinese population, Khek, 25% and Cantonese 24%. Teochiu, the fourth largest group comprising 7%, have been combined with the Hokkien in calculating rates because of their close cultural affinity. The major census grouping of Malays comprises 88% Malay, 10% Indonesian and 2% aboriginal peoples, including Temiar. Among the

Indian group, 81% are Tamil and the rest small groups of Telugu, Malayali, Punjabi, Pakistani and Ceylonese. The small groups of " others " has its 2 largest components in Eurasians and Europeans (cf. Table II).

Hospital and biopsy records provide only general ethnicity of patients and so a special survey was made of the 312 cases in Selangor to establish specific ethnicity, as well as ethnic ancestry, place of birth of the patient and ancestors (as far back as grandparents), and length of residence in states and countries. Interviews were completed for 192 cases; addresses were inadequate to find in 22 and 98 could not be traced, nor any suitable substitute informant. There was little difference in the proportion of cases located according to the year of diagnosis: as many cases were found for 1968 as for 1972. Not included with the 312 Selangor cases were 9 who were found at interview to have recently moved into the state for purposes of treatment. They were counted as residents of other states.

The 192 cases with completed interviews formed the basis for calculating estimated rates (Table IV). Age-adjusted rates for the Chinese sub-ethnic groups were prepared by the indirect method using the total Chinese population of Selangor as standard (Hill, 1971). Because population figures by age and sex are not compiled for the sub-ethnic groups, a random sample of 12,000 Chinese returns from the 1970 census of Selangor was drawn by the Malaysian Department of Statistics, and accurate estimates of the population age structure by sex and place of birth were obtained. All the rates were adjusted in proportion to the total numbers of known male and female Chinese and Malay cases in Selangor.

TABLE IV.—*Estimated Incidence of Nasopharyngeal Carcinoma among Chinese and Malay Sub-ethnic Groups, Selangor,* 1968–72

| Sub-ethnic group | | 1970 population | No. of NPC cases interviewed | Crude rate per 100,000 population per year | Age-adjusted rates per 100,000 population per year (A)[1] | (B)[1] |
|---|---|---|---|---|---|---|
| Chinese | | | | | | |
| Hokkien and Teochiu* | M | 162811 | 29 | 6·0 | 7·0 | 10·0 |
| | F | 153284 | 9 | 1·8 | 2·0 | 2·9 |
| Khek | M | 95904 | 32 | 11·3 | 11·8 | 18·9 |
| | F | 96271 | 16 | 5·2 | 5·0 | 7·1 |
| Cantonese | M | 88961 | 45 | 17·1 | 15·0 | 24·5 |
| | F | 93905 | 28 | 9·3 | 7·9 | 12·0 |
| Hainanese | M | 18513 | 3 | 5·5 | — | — |
| | F | 16454 | 4 | 7·6 | — | — |
| Henghua | F | 1920 | 1 | — | — | — |
| Malays | | | | | | |
| Malay | M | 257751 | 5 | 0·6 | | |
| | F | 241362 | 2 | 0·2 | | |
| Indonesian | M | 28692 | 9 | 9·4 | | |
| | F | 27934 | 1 | 0·7 | | |

* Hokkien and Teochiu are combined because they are closely related culturally. In 1970 the Teochiu population in Selangor was 26011 males and 23921 females. During the 1968–72 period there were 6 male and 1 female cases of NPC among the Teochiu.

[1](A) Age-adjusted by the indirect method using total 1970 Chinese male/female population of Selangor as standards.

[1](B) Age-adjusted to the world population.

For example, 109 out of 184 Chinese male patients were interviewed and so the rates for male Chinese sub-groups were multiplied by a factor of 184/109. This assumes that those not interviewed were distributed within sub-ethnic groups in the same proportion as those actually interviewed. Eight cases which were not tabled were 2 Indian Tamil males, 3 other Indian males, one Ceylon Tamil male, one Pakistani female and one Temiar female.

The Selangor rates for Chinese follow the pattern observed elsewhere: highest among the Cantonese, least among the Hokkien and Teochiu, with the Khek in between. The rate for the Khek is of interest because this community can be considered as genetically northeastern Chinese who have been settled in southern China for about 700 years (Forrest, 1965). They appear to have assimilated little with the other much longer established southern Chinese and are a somewhat under-privileged group. They might therefore have been expected to have lower incidence rates of nasopharyngeal carcinoma than not only the Cantonese but also the Hokkien and Teochiu—if it is true that rates are generally low in the north and high in the south and if genotype were more important than environment in the aetiology of nasopharyngeal carcinoma.

The similarity of the pattern of rates in Selangor to those observed elsewhere lends support to its validity. However, a question which arises is that the differences in rates among Chinese sub-ethnic groups could in part be due to differential use of medical services. In Selangor there is no easy way of testing whether or not Cantonese with nasopharyngeal carcinoma are more or less likely to have their disease diagnosed by histopathology than the Hokkien or Khek. One aspect which is now under study is the relative use made of Western and Chinese medical services by the sub-ethnic groups. However, while preference for Chinese medicine might delay early diagnosis by histopathology, it is less likely to affect in general the ultimate numbers diagnosed. There is evidence, in hospital records, of nasopharyngeal carcinoma patients being referred by Chinese medical practitioners to Western practitioners and thence to histopathology. That many patients are in an advanced state of disease when first seen at hospitals suggests that they may be trying other modes of treatment first or delaying diagnosis out of fear.

To see if there might be a difference between Chinese sub-ethnic groups in Selangor in terms of relative accessibility to the centres of cancer diagnosis, the residential addresses of patients interviewed were classified by census district and compared with the ethnic distributions of the general population. The 20 census districts of the Klang Valley, containing all the medical centres, formed the group " close " to centres, while the other 11 districts in the state comprised the group " distant " from centres. The percentages of patients interviewed who were in the " distant " category were: Hokkien/Teochiu 24%, Khek 44% and Cantonese 15%, while the corresponding figures for the general populations were 28%, 22% and 10%. These data suggest that accessibility to centres of diagnosis was not a significant factor in the differential rates among Chinese sub-groups.

In Singapore, China-born immigrants were not found to have a significantly higher risk for nasopharyngeal carcinoma than Singapore-born Chinese (Shanmugaratnam and Tye, 1970). This also appears to be the case among Malaysian Chinese in Selangor, where the proportions of China-born cases by sub-ethnic group are similar to those in the general population (Table V). The age distributions of the Hokkien/Teochiu, Khek and Cantonese in Selangor have minor differences. The Cantonese in both sexes have slightly higher proportions in the 30–65 year age groups, the Khek rank second and the Hokkien/Teochiu the third most youthful.

Other items of information from the interview survey revealed that intermarriage was very rare between sub-ethnic

TABLE V.—*Percentage Born in China: Patients Interviewed and Total Populations in Selangor Compared*

| Ethnic group | | NPC patients interviewed (age-adjusted)* | Total population 1970 |
|---|---|---|---|
| Hokkien and Teochiu | M | 10·7 | 10·0 |
| | F | 9·2 | 9·4 |
| Khek | M | 7·5 | 7·4 |
| | F | 12·7 | 9·5 |
| Cantonese | M | 12·5 | 11·9 |
| | F | 18·4 | 14·1 |

* The percentages were age-adjusted to the total populations. For example, the percentage for male Hokkien and Teochiu cases was age-adjusted to the total male Hokkien and Teochiu population.

groups at any generation, and that the pattern of place of birth among generations conformed to that of the general population. There was also marked consistency between provincial origin of the China-born and their sub-ethnic group: Cantonese were born in Kwangtung, Hokkien in Fukien and so on.

One interesting aspect of the rates for Malays in Table IV is that most of these cases were in fact Indonesians. This leads us to speculate that Indonesian immigrants are possibly more cosmopolitan than local Malay and perhaps more likely to seek modern treatment. The small number of cases among Malay precludes much comment but the evidence from the Singapore Cancer Registry and the indication of the present study is that the rates for Malay are closer to those of the Indians than the Chinese.

### *Pathology*

Pathological specimens from the nasopharynx of the 192 cases for which detailed ethnicity was established were re-examined to see if there was any correlation between histological type and sub-ethnic group. There were 98 " strip-mucosal " biopsies and 94 punch biopsies. The biopsies had been processed in the usual manner and sections were stained with routine haematoxylin and eosin stains. Special stains such as reticulin, mucicarmine and PAS had been used only in certain cases.

Histologically we observed 85 squamous cell and 107 undifferentiated carcinomata. Criteria for classifying carcinomata were in conformity with those of Shanmugaratnam and Muir (1967). Of the 85 squamous cell cancers, there were 11 spindle cell and 2 clear cell variants. Among the undifferentiated types the majority of them (93) were of the alveolar type and the remainder of the syncytial type. It is pertinent here to reiterate that ultrastructural studies have demonstrated that the undifferentiated carcinomata of the nasopharynx are, in fact, squamous cell carcinomata.

There was no correlation between histological type and the 3 sub-ethnic groups of Hokkien/Teochiu, Khek and Cantonese. This finding agrees with that of Svoboda, Kirschner and Shanmugaratnam (1967) who found no association between tumour histology and ethnicity among Chinese and Caucasians. Further studies now in progress and using a larger series may help to shed more light on this aspect.

Acknowledgements are made to the Director, Institute for Medical Research, the Director, General Hospital, Kuala Lumpur, and the University of California, San Francisco, International Center for Medical Research, for research support. We would also like to thank Dr H. S. Ahluwalia, Professor N. K. Yong, Dr C. Panicker, Dr J. K. Lucas, Dr K. Prathap, Mr J. Ponnudurai and Miss Cheah Guat Lin for technical assistance in case searching, and Miss D. Z. Fernandez and Mr K. G. R. Nathan for provision of special census data.

### REFERENCES

AHLUWALIA, H. S. & DUGUID, J. B. (1966) Malignant Tumours in Malaya. *Br. J. Cancer*, **20**, 12.

FORREST, R. A. D. (1965) The Southern Dialects of Chinese. In *The Chinese in Southeast Asia.* 2nd Edn. V. Purcell. London: Oxford University Press.

HILL, A. B. (1971) *Principles of Medical Statistics*, 9th Edn. London: Oxford.

HO, J. C. H. (1972) Nasopharyngeal Carcinoma. In *Advances in Cancer Research*. Eds. G. Klein and S. Weinhouse. New York: Academic Press.

KANNAN KUTTY, M. & BALASEGARAM, M. (1972) Malignant Tumours in West Malaysia. *Jl R. Coll. Surg. Edinb.*, **17**, 102.

MARSDEN, A. T. H. (1958) The Geographical Pathology of Cancer in Malaya. *Br. J. Cancer*, **12**, 161.

SHANMUGARATNAM, K. (1971) Studies on the Etiology of Nasopharyngeal Cancer. In *International Review of Experimental Pathology*. Vol. 10. New York: Academic Press.

SHANMUGARATNAM, K. (1973) Cancer in Singapore—Ethnic and Dialect Group Variations in Cancer Incidence. *Singapore med. J.*, **14**, 69.

SHANMUGARATNAM, K. & MUIR, C. S. (1967) Nasopharyngeal Carcinoma Origin and Structure. In *Cancer of the Nasopharynx*. U.I.C.C. Monog. Series, vol. 1. Copenhagen: Munksgaard.

SHANMUGARATNAM, K. & TYE, C. Y. (1970) A Study of Nasopharyngeal Cancer among Singapore Chinese, with Special Reference to Migrant Status and Specific Community (Dialect Group). *J. chron. Dis.*, **23**, 433.

SVOBODA, D. J., KIRSCHNER, F. R. & SHANMUGARATNAM, K. (1967) The Fine Structure of Nasopharyngeal Carcinomas. In *Cancer of the Nasopharynx*. U.I.C.C. Monog. Series, vol. 1. Copenhagen: Munksgaard.